# PReS-FINAL-2152: Biomarkers as predictors of early inactive disease in children with juvenile idiopathic arthritis

**DOI:** 10.1186/1546-0096-11-S2-P164

**Published:** 2013-12-05

**Authors:** M Lavric, T Weinhage, J Klotsche, G Horneff, JP Haas, K Minden, D Foell

**Affiliations:** 1 Department of Paediatric Rheumatology and Immunology, University Hospital Muenster, Muenster, Germany; 2 Programmbereich Epidemiologie, Deutsches Rheuma-Forschungszentrum Berlin, Berlin, Germany; 3 Asklepios Kinderklinik St. Augustin gmbh, Sankt Augustin, Germany; 4 Kinder-Rheumaklinik, Garmisch-Partenkirchen, Germany; 5 Otto-Heubner Centrum, Rheumaambulanz, Universitätsmedizin Berlin-Charité, Berlin, Germany

## Introduction

The outcome of children with juvenile idiopathic arthritis (JIA) has improved over the last decades. However, the disease course is still not easy to predict, especially based on traditional clinical factors. In the German prospective controlled observational multicentre study ICON-JIA (Inception Cohort Of Newly-diagnosed patients with JIA) patients with diagnosis of JIA <12 months are included for long-term observation to improve the prediction and understanding of JIA outcomes.

## Objectives

To analyse biomarkers (collected in the early phase (0-3 months) that may predict inactive disease within 12 months in JIA patients treated in specialized centres.

## Methods

ICON comprised an assessment by patients and parents via standardized questionnaires and clinical examinations by paediatric rheumatologists. Sera were collected from 191 children (132 girls, 59 boys), with JIA diagnosis based on the ILAR classification with a median age of 5 years (range = 1 to 17). At inclusion into the study, the disease duration since diagnosis was less than 6 months. All sera collected at inclusion and after 3 months were tested for biomarkers MRP8/14 and S100A12. Furthermore, 68 sera collected at inclusion were also assayed for CRP and ESR as well as the cytokines IL-12p70, IFN-gamma, IL-17A, IL-2, IL-10, IL-9, IL-22, IL-6, IL-13, IL-4, IL-5, IL-1 beta, and TNF-alpha.

## Results

After a 12 months follow-up, 106 children were diagnosed with inactive and 85 children with active disease. Of the biomarkers measured in sera collected at inclusion, only a higher ESR at baseline (median 27 vs. 19 mm/h, p = 0.013) was significantly associated with a persistently active disease at 12 months follow-up. While none of the cytokine levels revealed significant differences, both MRP8/14 (median 1,145 vs. 815 ng/ml, p = 0.041) and S100A12 levels (120 vs. 87 ng/ml, p = 0.032) in sera collected after 3 months of specialized care were significantly higher in patients who did not reach inactive disease status after 12 months. ROC analyses for MRP8/14 (area under curve, AUC 0.618, 95% CI 0.510-0.726, p = 0.041) and for S100A12 (AUC 0.651, 95% CI 0.514-0.717, p = 0.033) revealed a moderate but robust predictive power of these biomarkers.

## Conclusion

Markers of inflammation may help to assess the probability to achieve inactive disease within 12 months of specialised care in patients with early JIA. Markers of innate immune activation, S100A12 and MRP8/14, analysed after three months of specialized care, may potentially predict whether patients will reach inactive disease within one year of treatment.

## Disclosure of interest

None declared.

